# The Dawn of next generation DNA sequencing in myelodysplastic syndromes- experience from Pakistan

**DOI:** 10.1186/s12864-021-08221-w

**Published:** 2021-12-16

**Authors:** Nida Anwar, Faheem Ahmed Memon, Saba Shahid, Muhammad Shakeel, Muhammad Irfan, Aisha Arshad, Arshi Naz, Ikram Din Ujjan, Tahir Shamsi

**Affiliations:** 1 Department of Hematology and Genetics, National Institute of Blood Diseases and Bone Marrow Transplantation, Karachi, Pakistan; 2 Department of Pathology, Liaquat University of Medical & Health Sciences, Jamshoro, Sindh Pakistan; 3 Jamil-ur-Rahman Center for Genome Research, Dr. Panjwani Center For Molecular Medicine & Drug Research, International Center for Chemical and Biological Sciences, University of Karachi, Karachi, Sindh Pakistan; 4 Jamil-ur-Rahman Center for Genome Research, Dr. Panjwani Center For Molecular Medicine & Drug Research, University of Karachi, Karachi, Sindh Pakistan

**Keywords:** Myelodysplastic syndromes, Next generation sequencing, Gene analysis, Mutation, Pakistan

## Abstract

**Background:**

Myelodysplastic syndromes (MDS) are clonal disorders of hematopoietic stem cells exhibiting ineffective hematopoiesis and tendency for transformation into acute myeloid leukemia (AML). The available karyotyping and fluorescent in situ hybridization provide limited information on molecular abnormalities for diagnosis/prognosis of MDS. Next generation DNA sequencing (NGS), providing deep insights into molecular mechanisms being involved in pathophysiology, was employed to study MDS in Pakistani cohort.

**Patients and methods:**

It was a descriptive cross-sectional study carried out at National institute of blood diseases and bone marrow transplant from 2016 to 2019. Total of 22 cases of MDS were included. Complete blood counts, bone marrow assessment and cytogenetic analysis was done. Patients were classified according to revised WHO classification 2016 and IPSS score was applied for risk stratification. Baseline blood samples were subjected to analysis by NGS using a panel of 54 genes associated with myeloid malignancies.

**Results:**

The median age of patients was 48.5 ± 9.19 years. The most common presenting complaint was weakness 10(45.45%). Cytogenetics analysis revealed abnormal karyotype in 10 (45.45%) patients. On NGS, 54 non-silent rare frequency somatic mutational events in 29 genes were observed (average of 3.82 (SD ± 2.08) mutations per patient), including mutations previously not observed in MDS or AML. Notably, two genes of cohesin complex, RAD21 and STAG2, and two tumor suppressor genes, CDKN2A and TP53, contained highest number of recurrent non-silent somatic mutations in the MDS. Strikingly, a missense somatic mutation p.M272Rof Rad21 was observed in 13 cases. Overall, non-silent somatic mutations in these four genes were observed in 21 of the 22 cases. The filtration with PharmGKB database highlighted a non-synonymous genetic variant rs1042522 [G > C] located in the TP53. Genotype GG and GC of this variant are associated with decreased response to cisplatin and paclitaxel chemotherapy. These two genotypes were found in 13 cases.

**Conclusion:**

Sequencing studies suggest that numerous genetic variants are involved in the initiation of MDS and in the development of AML. In countries like Pakistan where financial reservation of patients makes the use of such analysis even more difficult when the availability of advanced techniques is already a prevailing issue, our study could be an initiating effort in adding important information to the local data. Further studies and large sample size are needed in future to enlighten molecular profiling and ultimately would be helpful to compare and contrast the molecular characteristics of Asian versus global population.

**Supplementary Information:**

The online version contains supplementary material available at 10.1186/s12864-021-08221-w.

## Introduction

Myelodysplastic syndrome (MDS) constitutes a heterogeneous group of clonal hematopoietic disorder of stem cells characterized by blood cytopenias in the presence of morphological dysplasia and tendency for leukemic transformation [1, 2]. Understanding the nature of disease is imminent for analysis of various clinical, biological and genomic factors involved in variability of disease evolution from indolent cytopenias to aggressive leukemic progression [1]. Previously many pathogenic mechanisms have been proposed including clonal, immune and genetic leading increased apoptosis [2]. However, during the past decade various sequencing technologies have played a major role in revealing major insights of disease pathogenesis unfolding disease genomics. Efforts are being made to study clinical implications of the disease genetics in terms of prognosis and treatment [3]. Heterogeneous clinical and risk-adapted treatments have along these lines been produced, considering identified genomic mutational profiling. Hereditary and epigenetic variations form the standard of myeloid neoplasia advancement and in spite of the level of dysplasia and impact rates yet being the primary highlights for the WHO classification, a lot of information has turned out to be accessible on repeating transformations in MDS, fundamentally because of massive parallel sequencing strategies [1]. Working in third world country like Pakistan, where we have limited resources for health, education and scientific research, implementation of genomics disciplines are believed to be valuable tools to advance knowledge as well as improve health risk identification, diagnoses, treatment and prevention [2]. Next-generation sequencing (NGS) now considers synchronous sequencing and investigation of numerous qualities, as opposed to the more relentless single-quality examine approach, and has quickened the revelation of pathogenic transformations in MDS, Myeloproliferative neoplasms (MPNs), and acute myeloid leukemia (AML). Therefore, NGS has extraordinary potential as a component of the analytic calculation in these disorders, especially in challenging cases without cytogenetic markers of clonality [3]. Around 80 to 90% of patients with MDS harbor disease-associated gene mutations, frequently in spliceosomal genes and epigenetic regulators. The most commonly mutated genes in MDS include ASXL1, TET2, RUNX1, NRAS, SF3B1, SRSF2, and TP53. Gene mutations carry prognostic and therapeutic significance, and there have been several proposed prognostic models in MDS based on mutation status [4–6]. Mutations in TP53, EZH2, ETV6, RUNX1, and ASXL1 are indicators of poor overall survival in patients with MDS, autonomous of other established risk factors, and SF3B1 mutations confer a better clinical outcome. Mutations in TET2 have additionally been appeared to predict response to hypomethylating agents, particularly in cases without a concomitant ASXL1 mutation [7–10]. Many studies have been coordinated to this expanded information of quality changes in our comprehension of MDS pathogenesis and into clinical practice, which is significantly more emphasized by the ongoing development of high-throughput genomics and suggests that the mutational status of multiple gene targets could better predict the clinical outcome in MDS [8, 9]. The purpose of the present study was to evaluate various genetic mutations in the disorder in our patients using revolutionized technologies for genome analysis in third world country like Pakistan.

## Results

This retrospective study involves categorical investigation of genetic alterations in 22 MDS cases of the second highest populated region of South Asia (Pakistan) through deep massively parallel DNA sequencing using a targeted TruSight myeloid sequencing panel. This panel is used for detecting the somatic variations in genes commonly mutated in myeloid malignancies. The targeted coding and non-coding regions were covered equally where the median depth of coverage for non-coding and coding variants was 4999x and 4920x respectively. The low quality variants with parameters of QUAL< 50, DP < 30, and GQ < 20, were filtered out to minimize the potential variants due to sequencing artifacts. As a result, 265 variants in 44 genes were obtained, with an average of 77.09 variants (SD ± 7.39), and median of 75.5 variants per sample.

The genomic locations and their functional impact of the identified mutations were obtained by the annotation with ANNOVAR (detailed in Table 1). It was noted that the number of mutated non-synonymous (nonsyn) sites was higher than mutated synonymous (syn) sites, and the nonsyn/syn ratio was found as 1.15 which is higher than previously reported ratio of germline missense to silent variants in the South Asian populations [11]. For normalization and comparison, the nonsyn/syn ratio was also determined in PJL (Punjabi Lahore, Pakistan) healthy individuals of 1000 Genomes Project using the genetic variants within the same genomic regions as sequenced in this study. The ratio in healthy individuals was found as 0.88, which is 0.765 times the ratio in MDS cases of present study. Further analysis showed that the higher proportion of novel/rare nonsynonymous SNVs in present study MDS cases than in healthy individuals of 1000 Genomes Project was responsible for higher nonsyn/syn ratio in the study genes. There were 37 nonsynonymous SNVs either not present or had < 0.1% alternate allele frequency in 1000 Genomes and gnomAD_exome projects, whereas this number was 19 for synonymous SNVs. The higher nonsyn/syn in the MDS patients is persistent with previous reports [6, 12].

**Table 1:** The functional annotation genetic variants by Annovar

Genomic region	No. of variants
Exonic	127
Intronic	122
Splicing region	01
Downstream	02
UTR5	02
UTR3	11
**Functional Impact**	
Nonsynonymous	62
Synonymous	54
Stop-gain	02
Splicing	01
Frameshift insertion	07
Non-frameshift insertion	01
Non-frameshift deletion	01

**Fig. 1:**
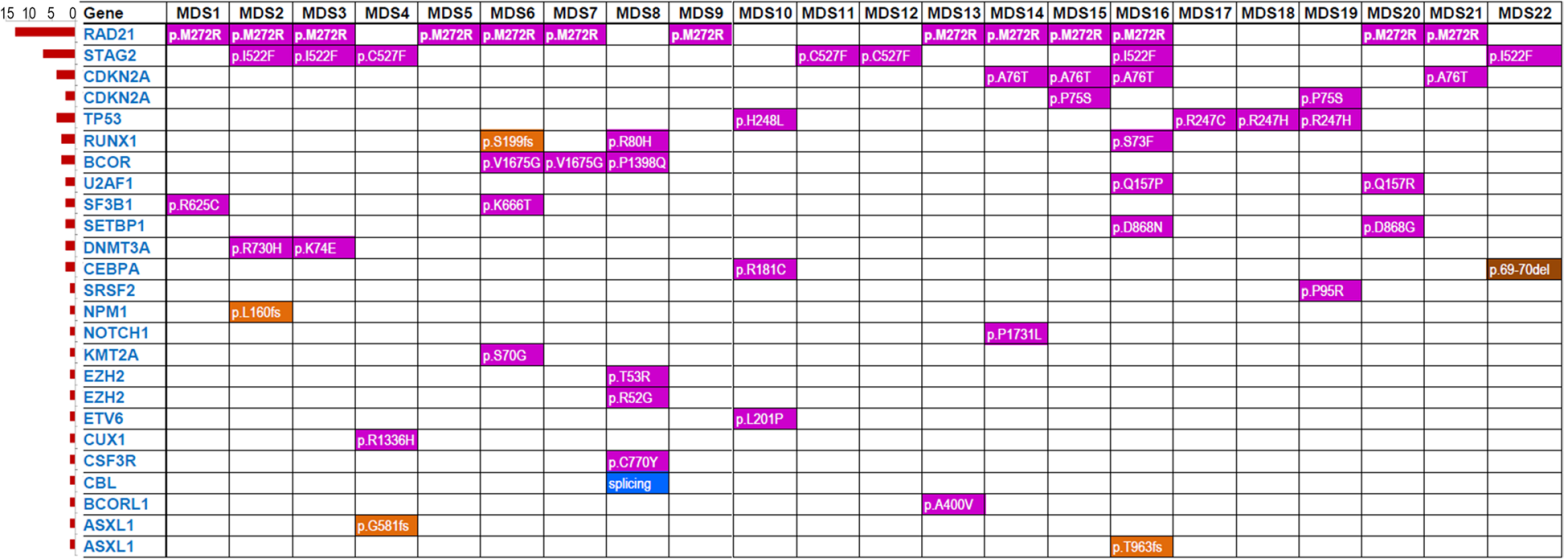
Frequency distribution of non-silent somatic mutations within the MDS patients of this study. Notably, *RAD21* and *STAG2* genes were found highest number of recurrent somatic mutations, 13 and 7 respectively

To explore potential deleterious impact of identified variants, emphasis was given to rare variants given the MDS is a rare disorder. The variants either not present or having alternate allele frequency < 1% in all the populations of public databases including 1000 Genomes Project and gnomAD_exome projects were retained. This resulted in 120 rare frequency mutational events (average 13.318 (SD ± 4.07) mutations per patient) in 38 genes including 02 stopgain, 42 nonsynonymous, 21 synonymous, 01 canonical splicing, 01 downstream,03 3′ untranslated region (UTR), and 34 intronic SNVs, and 07 frameshift insertions, 01 non-frameshift insertion, 01 non-frameshift deletion, and 07 intronic deletions (Supplementary Table S1). Furthermore, excluding the intronic, intergenic, synonymous, upstream/downstream and UTR mutations, there were 54 non-silent rare frequency mutations in 29 genes where three patients had one non-silent mutation and nineteen patients had more than one non-silent mutations, average 3.82 (SD ± 2.08) non-silent mutations per patient (Fig. 1).

Given that NGS was performed on the DNA isolated from peripheral blood containing both the normal leukocytes and blast cells, we applied a bit stringent unanimous cut-off 0.35 on variants allelic fraction (VAF) for all patients for discriminating probable somatic mutations from the germline. This probe decomposed 54 rare non-silent variants into 37 somatic non-silent mutations in 22 genes (Supplementary Table S2) and 17 germline non-silent mutations in 15 genes (Supplementary Table S3), representing multiple underlying mechanisms involved in pathophysiology of MDS in this cohort. Among the somatic mutations, 8 mutations were recurrent being found in more than one patients. There were 6 MDS cases containing one and 16 cases containing more than one somatic mutations each. Strikingly, it was noticed that the non-synonymous somatic mutation rs752628932 in highly conserved region (exon 8) of RAD21 (c.T815G; p.M272R) was present in 13 out of 22 cases (59%cases of this small cohort). The VAF of this substitution mutation was observed ranging from 0.172 to 0.262 indicating slightly variable time of origin in the patients. The other recurrent somatic mutations included nonsynonymous SNV c.A1564T:p.I522F in highly conserved region of STAG2observed in four cases, nonsynonymous SNV c.G1580T:p.C527F in the same conserved region of STAG2 observed in three cases, and nonsynonymous SNV c.G226A:p.A76T in CDKN2A observed in four cases. The two STAG2 mutations (p.I522F and p.C527F) were observed in different patients. Among the germline mutations, two mutations were recurrent found in three cases each. These included a protein truncating SNV c.C1894T: p.R632X in highly conserved region of ASXL3, and a nonsynonymous SNV c.T1604C:p.M535T in highly conserved region of KIT. There were 13 cases having both germline non-silent mutation and somatic non-silent variants, however, no statistically significant correlation was observed between the number of predisposing germline mutations and the somatic mutations within the cases (*P* > 0.05). For example, the MDS2 and MDS16 cases contained four and seven non-silent somatic mutations respectively, whereas these did not contain a predisposing germline non-silent mutation. Likewise, MDS3 and MDS4 had 3 and 4 germline predisposing mutations respectively, whereas both these cases had 3 somatic mutations each.

Filtration of the variants with ClinVar database highlighted the presence of four pathogenic variants associated with hematological neoplasms. These included a recurrent missense SNV rs121913250 (p.G12S) in highly conserved region of NRAS associated with acute myeloid leukemia and juvenile myelomonocytic leukemia, found in three cases; a frameshift insertion p.L160fs in NPM1, associated with myelodysplastic syndrome progressed to acute myeloid leukemia, found in one case; a missense SNV p.R730H in highly conserved region of Dnmt3a, associated with acute myeloid leukemia, myelodysplastic syndrome, lung adenocarcinoma, and inborn genetic diseases, found in one case; and a splicing SNV in CBL (exon8:c.1096-2A > T) found in one case. Furthermore, filtration in PharmGKB database showed the presence of a missense SNV rs1042522[G > C] in TP53 where GG genotype was found in two and GC in eleven cases. The GG and GC genotypes are associated with decreased response to cisplatin and paclitaxel chemotherapy.

## Discussion

Next generation DNA sequencing and analysis of hematological neoplasms including MDS has provided several remarkable advantages in the diagnosis, prognosis, and personalized therapeutic choices [13, 14]. In this retrospective study, the MDS patients were recruited in a clinical diagnostic setup for performing ultra-deep(~5000x) targeted genes sequencing by using Illumina myeloid sequencing panel. The study provides clinico-pathological significance of the identified potential pathogenic non-silent genetic alterations in MDS in the Pakistani population. To the best of our knowledge, the current study is the first to report genetic variations in MDS from this region of South Asia using the NGS technology.

By applying a schematic bioinformatics approach, 265 genetic variants were identified which included 224 single nucleotide point mutations and 41 small indels in the targeted exonic and non-exonic regions in a small cohort of 22 MDS patients. A variant was considered as novel if it was not found in dbSNP151, gnomAD, and ClinVar databases. For assessing potential pathogenicity of the identified rare mutations (minor allele frequency < 1% in global populations), the ACMG criteria of several bioinformatics tools was employed [15]. Three in silico tools including SIFT, Polyphen2, and CADD along with the conservation scores were used to assess pathogenic impact of non-synonymous variants as described previously [16]. The scores generated by these tools were strong and convincing enough to suggest the possible pathogenicity of the variants in respective cases.

The higher nonsyn/syn ratio in the MDS cases is the indication of excessive mutation rate and/or positive selection at the non-synonymous sites, which is in-correlation with previous studies on cancers [12, 17]. By using the prioritization approach of multiple in silico tools, at least one pathogenic/deleterious non-silent predisposing mutation was detected in all the 22 cases (average 3.82 mutations per patient). The gene mutations in multiple genes represents diverse underlying mechanisms in the pathophysiology of MDS in this cohort. Notably, the missense somatic mutation p.M272R in RAD21 was observed in 13 patients that constitute 59% of this small cohort. RAD21 is the component of cohesin complex and is involved in the repair of DNA double-strand breaks as well as in chromatid cohesion during mitosis [18]. Following the RAD21, the second highest number of non-silent mutations were observed in STAG2, where two recurrent mutations (p.I522F and p.C527F) were observed in seven different samples as a whole (31.8% patients). The STAG2 is a subunit of the cohesin complex which regulates the separation of sister chromatids during cell division (Gene Cards STAG2, 2020). Collectively, the two genes RAD21and STAG2 contained mutations in 17 samples which constituted 77.27% patients of the cohort, and belong to cohesin complex. The presence of highly recurrent non-silent mutations in genes of cohesin complex denotes the underlying pathophysiological role of the impairment of DNA breaks repair and proper segregation of genetic material during the mitotic division of the hematopoietic cells in the MDS cases of this study. Previously, the mutations in RAD21 and STAG2 were accounted for de novo AML in 6.9% of the unrelated cases [19]. In the Cancer Genome Atlas (TCGA), the presence of mutations within genes encoding the cohesin complex has been reported in 13% of the AML patients [20]. According to Tsai et al. (2017) [19], the AML patients with mutations in cohesin complex genes presented better overall survival (OS), and disease free survival (DFS) than those without cohesin complex genes mutations, whereas, according to Thol et al. [21], overall survival, relapse-free survival, and complete remission rates were not influenced by the presence of cohesin mutations. In the present study, 41% of the cases with cohesin complex gene mutations expired during the course of follow up.

Among the other oncogenic mechanisms potentially involved in the MDS patients included the mutations in tumor suppressor genes. Tumor suppressor gene CDKN2A containing mutations in five samples, and TP53 containing mutations in four samples, collectively constituted 36.36% cases in the cohort. The CDKN2A encodes a tumor suppressor protein from alternate open reading frame (ARF) transcript and functions as a stabilizer of the tumor suppressor protein p53 by interacting with the E3 ubiquitin-protein ligase MDM2, a protein responsible for the degradation of p53 (Refseq CDKN2A, 2020). The TP53 encodes a tumor suppressor protein p53 which contains three domains i.e., transcriptional activation, DNA binding, and oligomerization domains. The encoded protein responds to diverse cellular stresses to regulate expression of target genes, thereby inducing cell cycle arrest, apoptosis, senescence, DNA repair, or changes in metabolism [22].

From the filtration with ClinVar database, four pathogenic mutations associated with hematological malignancies including recurrent p.G12S in NRAS found in three cases; a frame shift insertion p.L160fs in NPM1 found in one case, a missense SNV p.R730H in DNMT3A found in one case, and a splicing SNV in CBL fond in one case were noted. This represents additional mutations, other than the associated variants, being involved in the pathophysiology of MDS in the patients of this study.

## Conclusion

This study presents a comprehensive analysis of somatic and germline mutations in MDS from a South Asian country (Pakistan) using next-generation DNA sequencing technology. The spectrum of potential pathogenic mutations identified in this study strongly suggests that mutations in cohesin complex genes and tumor suppressor genes predominate the underlying mechanisms in MDS. The identified rare and novel deleterious mutations would add to the repertoire disease causing mutations. This study also presents the feasibility and employment of sequencing the targeted genes in challenging and complex MDS cases. The limiting factors of this study include retrospectively inclusion of small cohort size from a single medical center. The fruitfulness of the novel findings of present study can be increased by validation in replicate studies of larger cohort with different time scales. Nevertheless, the findings provide an assessment of predisposing detrimental mutations in MDS in this region and its utility in clinical settings.

## Material & Methods

### Ethical consideration and consent statement

The current study was approved by the ethical review board of the National Institute of Blood Diseases and Bone Marrow Transplantation (NIBD) under the ethical protocol approval no. NIBD/RD-175/16–2015, and was performed according to the ethical guidelines of the Declaration of Helsinki. For this study, a total of 22 MDS cases, confirmed by the hematologists on the basis of patients’ clinical laboratory investigations, were enlisted between March 2016 and Dec 2019. The median age of the patients was 48.5 ± 9.19 years. The clinical details of the study subjects, and cytogenetic analysis are given in Table 2. A pre-approved written informed consent was obtained from all the studied participants before the samples collection. Whole blood sample was collected from the enrolled patients in the EDTA tube and stored at 4 °C till further process.

**Table 2:** Clinical characteristics of myelodysplastic syndrome patients

Variable	
Number of patients (N)	22
Age (Median and SD)	48.5 ± 9.19
Male to Female ratio	3:01
Hemoglobin (Hb) (mg/dl), (Median and SD)	8.7 ± 1.6
Total leucocyte count (TLC) (*10^9/l), (Median and SD)	9.7 ± 14.3
Platelet count (*10^9/l), (Median and SD)	90 ± 77.6
Absolute neutrophils count (ANC) (*10^9/l), (Median and SD)	1.55 ± 0.91
**MDS Category**	
**Low Risk**	
MDS-MLD	9 (41%)
MDS-SLD	2 (9.09%)
MDS-SLD-RS	1 (4.54%)
MDS-U	1 (4.54%)
**High Risk**	
MDS-EB2	7 (31.80%)
MDS-EB1	1 (4.54%)
MDS-AML	1 (4.54%)
**Cytogenetics**	
Normal karyotype	12 (54.54%)
Del5q	3 (13.60%)
Del7q	3 (13.60%)
Complex karyotype	3 (13.60%)
Monosomy 20	1 (4.54%)
**Total number of mutations**	
No mutation	10 (45.45%)
Mutations	12 (54.54%)
**Mutations in high risk MDS group**	**7 (58%)**
p.Gly12Ser NRAS	3 (25%)
p.Pro384Leu (het) RunX1	1 (8.3%)
ASXL1 C2077C > T,BCORL1 C.331 T > C,TET2 c.1064G > A	1 (8.3%)
BCORL1 c.3315 T > C,EZH2c.553G > C	1 (8.3%)
p. Arg107His RunX1,p.Pro75His CDKN2A,p.Thr358Pro GATA2	1 (8.3%)
**Mutations in low risk MDS group**	**5 (42%)**
p.Ile428Thr(het) RunX1	1 (8.3%)
p. Pro75Leu CDKN2A	1 (8.3%)
Tet-2 c5162 T > G mutation	1 (8.3%)
p.Gln1039Ter (het) ASXL1	1 (8.3%)
DNMT3A(c.2645 G > A), Arg 882 His,Npm1 c.859–860 ins TCTG p. Trp 288.Cysfs Ter 12	1 (8.3%)

### DNA extraction

Isolation of the genomic DNA was carried out from the whole blood by using the QIAamp DNA Blood Mini Kit (QIAGEN, Hilden, Germany). The quality of the isolated genomic DNA was evaluated using agarose gel electrophoresis and the concentration of DNA was estimated by Qubit fluorometer using DNA High sensitivity kit (Invitrogen, Thermo Fisher Scientific, USA).

### Myeloid sequencing panel

TruSight myeloid sequencing panel (Illumina, San Diego, CA, USA) is designed to sequence targeted region, coding regions and exonic hotspots, of 54 genes harbors frequent somatic variations. The exonic region of fifteen genes i.e., CDKN2A, BCOR, BCORL1, CEBPA, STAG2, DNMT3A, CUX1, IKZF1, ZRSR2, RUNX1/AML1, PHF6, EZH2, RAD21, ETV6/TEL, and KDM6A while the exonic hotspots of 39 genes i.e., ATRX, ASXL1, BRAF, CBL, CBLB, CBLC, CALR, CSF3R, FLT3, JAK2, GATA1, GATA2, GNAS, KIT, FBXW7, HRAS, KRAS, NRAS, IDH1, IDH2, JAK3, KMT2A/MLL, NPM1, NOTCH1, MYD88, MPL, PTEN, PDGFRA, SETBP1, PTPN11, SMC1A, SF3B1, SRSF2, SMC3, U2AF1, TET2, WT1, and TP53 sequenced using Illumina platform. This panel comprised of 568 amplicons covering the target region of interest ~ 250 bp in length.

### DNA libraries preparation

DNA paired end libraries were constructed from the genomic DNA (50 ng) using TruSight Myeloid Sequencing Panel as per the kit protocol. First the customized probes hybridized to the upstream and downstream of the targeted region of interest and then the unbound oligos were removed using subsequent washing steps. The hybridized upstream oligos were extended by DNA polymerase to the bound downstream hybridized oligos and ligation was carried out by DNA ligase. This extension step covers the targeted region. The adapters and indexes were added and followed by amplification of the product using PCR. The amplified product was then purified by using Agencourt Ampure XP beads. The concentration of the purified library containing the target region of interest was estimated by using HS DNA Qubit kit. The library was then normalized (equal representation of each library in the pool library) as per manufacture protocol. The normalized library was then pooled i.e., 5 uL from each library was added to the single tube. The pooled library was diluted with HT1 buffer (6.0 uL of pooled library and 694 uL HT1 buffer) and then denatured at 92 oC. The diluted library was added to the V2 (MS-102-2002) sequencing cartridge kit [6] and then subjected to the next generation sequencing using MiSeq Illumina platform.

### Data analysis

The variant calling was carried out by using standard pipeline [23]. The Burrows–Wheeler Aligner (BWA-MEM) algorithm was used to align the short DNA sequenced reads with reference human genome hg19 [24]. Samtools package was used to convert the sequence alignment/map files (SAM) file to binary alignment/map files (BAM) [25]. The PCR duplicates were removed by using PICARD tool http://picard.sourceforge.net and Genome Analysis Tool Kit (GATK) pipeline used as the best practice for the base quality score recalibration (BQSR), indels and variant calling [26]. Such variants having GQ > =20 20, QUAL > = 50 and rare variants (variant allele frequency < 1%) in either 1000 Genomes Project and/or gnomAD_genome were selected for downstream analysis [27]. We adopted a prioritization approach employing multiple in silico tools to find out the deleterious and/or pathogenic variants as suggested by American College of Medical Genetics and Genomics [15]. The functional consequences of the obtained genetic variants were carried out by using two annotation tool i.e., ANNOVAR [28] and Ensembl’s annotation algorithm Variants Effect Predictor (VEP) [29]. We carried out the SIFT and Polyphen2 tools and CADD phred scores of the rare variants to find out the deleterious impact of missense variants [30].

A parsimony-guided unsupervised functional impact predictor tool ParsSNP was used to determine the biologically active driver mutations over the inactive passenger mutations. The expectation maximization framework was employed to find key genetic variants. This approach explains tumor incidence independently without using the predefined training labels/datasets which may be potential source of enrichment biases [31]. ClinVar database was searched to find out the pathogenic [32] impact of variants that are previously reported with myeloid malignancies. The protein-protein interactions (of prioritized genes) was determined using the online STRING database [33]. Furthermore, we searched the variants from manually curated pharmGKB database [34] to determine the role in chemotherapeutic agents against leukemia.

## Supplementary Information

**Additional file 1 Supplementary Table S1.** Rare Frequency varints in MDS Patients.

**Additional file 2 Supplementary Table S2.** Somatic mutations in MDS Patients.

**Additional file 3.**

**Additional file 4.**

## Data Availability

Bioproject accession number obtained was PRJNA725337, with a release date of 2021-05-04, and the SRA records will be accessible with the following link: https://www.ncbi.nlm.nih.gov/sra/PRJNA725337
